# Back to the future: Transplanting the chloroplast TrxF–FBPase–SBPase redox system to cyanobacteria

**DOI:** 10.3389/fpls.2022.1052019

**Published:** 2022-11-28

**Authors:** Raquel García-Cañas, Francisco J. Florencio, Luis López-Maury

**Affiliations:** ^1^ Instituto de Bioquímica Vegetal y Fotosíntesis, Universidad de Sevilla- CSIC, Sevilla, Spain; ^2^ Departamento de Bioquímica Vegetal y Biología Molecular, Facultad de Biología, Universidad de Sevilla, Sevilla, Spain

**Keywords:** redox regulation, Calvin Benson Bassham cycle, thioredoxin, cyanobacteria, FBPase, SBPase

## Abstract

Fructose-1,6-bisphosphatase (FBPase) and sedoheptulose-1,7-bisphosphatase (SBPase) are two essential activities in the Calvin–Benson–Bassham cycle that catalyze two irreversible reactions and are key for proper regulation and functioning of the cycle. These two activities are codified by a single gene in all cyanobacteria, although some cyanobacteria contain an additional gene coding for a FBPase. Mutants lacking the gene coding for SBP/FBPase protein are not able to grow photoautotrophically and require glucose to survive. As this protein presents both activities, we have tried to elucidate which of the two are required for photoautrophic growth in *Synechocystis* sp PCC 6803. For this, the genes coding for plant FBPase and SBPase were introduced in a SBP/FBPase mutant strain, and the strains were tested for growth in the absence of glucose. Ectopic expression of only a plant SBPase gene did not allow growth in the absence of glucose although allowed mutation of both *Synechocystis*’ FBPase genes. When both plant FBPase and SBPase genes were expressed, photoautrophic growth of the SBP/FBPase mutants was restored. This complementation was partial as the strain only grew in low light, but growth was impaired at higher light intensities. Redox regulation of the Calvin–Benson–Bassham cycle is essential to properly coordinate light reactions to carbon fixation in the chloroplast. Two of the best characterized proteins that are redox-regulated in the cycle are FBPase and SBPase. These two proteins are targets of the FTR-Trx redox system with Trx *f* being the main reductant *in vivo*. Introduction of the TrxF gene improves growth of the complemented strain, suggesting that the redox state of the proteins may be the cause of this phenotype. The redox state of the plant proteins was also checked in these strains, and it shows that the cyanobacterial redox system is able to reduce all of them (SBPase, FBPase, and TrxF) in a light-dependent manner. Thus, the TrxF–FBPase–SBPase plant chloroplast system is active in cyanobacteria despite that these organisms do not contain proteins related to them. Furthermore, our system opens the possibility to study specificity of the Trx system *in vivo* without the complication of the different isoforms present in plants.

## Introduction

Cyanobacteria are eubacteria that can perform oxygenic photosynthesis similar to higher plants and the evolutionary precursors of plant chloroplast. Because of their abundance in nature and their ability to fix carbon, cyanobacteria play an essential role in the global carbon cycle and are responsible for an important fraction of CO_2_ fixation in the biosphere (Falkowski et al., 1998).CO_2_ fixation is performed through the Calvin–Benson–Bassham Cycle (CBB). In this cycle, fructose-1,6-bisphosphatase (FBPase; EC 3.1.3.11) and sedoheptulose-1,7-bisphosphatase (SBPase; EC 3.1.3.37) are two essential activities (Bassham et al., 1954). FBPase transforms fructose-1,6-bisphosphate into fructose-6-phosphate and inorganic phosphate, whereas SBPase catalyzes the hydrolysis of sedoheptulose-1,7-bisphosphate into sedoheptulose-7-phosphate and inorganic phosphate. In photosynthetic organisms, FBPase is necessary in CBB, the oxidative pentose pathway (OPP), and gluconeogenesis. On the other hand, SBPase is only involved in the regeneration phase of the CBB. Although the FBPase and SBPase activities are separated in two different enzymes in eukaryotic photosynthetic organisms, in cyanobacteria, a single protein presents both activities. Most cyanobacteria also contain an additional enzyme that only exhibits FBPase activity (Udvardy et al., 1982; Tamoi et al., 1996; Tamoi et al., 1998; Tamoi et al., 1999; Nakahara et al., 2003). Cyanobacterial proteins have a different evolutionary origin to plant FBPase and SBPase (Jiang et al., 2012; Gütle et al., 2016), which were acquired later during plant evolution (Sahrawy et al., 1996; Capitani et al., 2000; Jiang et al., 2012; Gütle et al., 2016; Mallén-Ponce et al., 2022).

In oxygen-photosynthetic organisms, several enzymes of the CBB cycle are redox-regulated in a light-dependent manner, and thioredoxins play a crucial role in this process (Balsera et al., 2014; Buchanan, 2016; Gurrieri et al., 2021). In cyanobacteria, early studies revealed the capacity of Trxs to activate cyanobacterial FBP/SBPase (Pelroy et al., 1976; Schmidt, 1980; Udvardy et al., 1982; Crawford et al., 1984) and also by the light/dark cycles (Pelroy et al., 1976; Crawford et al., 1984). Subsequently, it was proposed that FBP/SBPase was resistant to H_2_O_2_ and not redox-regulated (Tamoi et al., 1996; Tamoi et al., 1998). Other CBB enzymes such as phosphoribulokinase (PRK) and glyceraldehyde-3-phosphate dehydrogenase (GAPDH) have also been found to be regulated by the light/dark cycles in different photosynthetic organisms (including cyanobacteria) through their interaction with CP12, forming an inactive complex (Wedel and Soll, 1998; Kobayashi et al., 2003; Michelet et al., 2013; Gurrieri et al., 2021). Recently, the structures of the GAPDH/CP12/PRK complex and PRK from *Thermosynechococcus elongatus* BP-1 and *Synechococcus elongatus* PCC 7942 were solved and revealed the regulations of redox signaling for PRK and CP12 in cyanobacteria (McFarlane et al., 2019; Wilson et al., 2019; Yu et al., 2020).

Regulation of these enzymes allows the control and fine-tuning of the CBB by which organisms adapt to changing light conditions and/or day/night transitions. Plant CBB is finely tuned to adapt to these changes, and several enzymes of the cycle are regulated by multiple mechanisms (Cejudo et al., 2019). One of these layers of regulation is redox control of the enzymes. FBPase and SBPase are two of the best studied redox-regulated enzymes in the cycle (Balsera et al., 2014; Buchanan, 2016; Balsera and Buchanan, 2019). These two proteins are active in their reduced form, and the FTR-Trx system is responsible for their reduction in the light (Buchanan, 2016; Gütle et al., 2016; Naranjo et al., 2016; Geigenberger et al., 2017; Ojeda et al., 2017; Yokochi et al., 2019). Thioredoxins are small redox proteins (~12 kDa) that have been found in all organisms (Schürmann and Buchanan, 2008; Balsera and Buchanan, 2019) and are capable of reducing disulfide bridges in other proteins. The plant FTR-Trx system has a cyanobacterial origin and with most of its components [FTR, Trx *m*, Trx *x*, Trx *y*, and NADPH thioredoxin reductase C (NTRC)] being present in cyanobacteria (Buchanan, 2016; Geigenberger et al., 2017; Mallén-Ponce et al., 2022).

Here, we have complemented a SBP/FBPase mutant strain with plant SBPase and/or FBPase genes to elucidate which of these two enzymatic activities are essential for growth. We have found that both genes are needed for complementation; nevertheless, *Synechocystis* contains an additional genes coding for a FBPase. Furthermore, we were able to show that both plants chloroplast proteins were reduced by the cyanobacterial thioredoxin system although with reduced efficiency. Finally, we have also shown that introduction of TrxF1 increases enzymatic activity and growth of these complemented strains, demonstrating that TrxF1 is able to work in cyanobacteria, despite this protein not being naturally present in these organisms.

## Material and methods

### Strains and growth conditions

All *Synechocystis* strains used in this work were grown photoautotrophically on BG11C, mixotrophically in BG11C supplemented with 10 mM glucose or heterotrophically in BG11C with 10 mM glucose and 20 µM 3-(3,4-dichlorophenyl)-1,1-dimethylurea (DCMU) at 30°C under continuous illumination (5–500 µmol photon m^−2^ s^−1^) in a 250-ml Erlenmeyer flask containing 50 ml of media or under bubbling conditions as described in the figure legends. For plate cultures, medium was supplemented with 1% (wt/vol) agar. Kanamycin, nourseothricin, erythromycin, and spectinomycin were added to a final concentration of 50, 50, 10, and 5 µg ml^−1^, respectively. Experiments were performed using cultures from the logarithmic phase (0.6–1 OD_750nm_, 3–5 µg of chlorophyll ml^−1^; 1 OD_750nm_ is equivalent to 4 × 10^7^ cells ml^−1^). *Synechocystis* strains are described in **Table S1**. All *Synechocystis* strains were checked by PCR analysis for complete segregation of all copies of the chromosomes using the appropriated primers as indicated in the figures. All oligonucleotides used in this work are described in **Table S2**.

*E. coli* DH5α cells were grown in Luria Broth supplemented with ampicillin (100 µg ml^−1^), kanamycin (50 µg ml^−1^), and spectinomycin (50 µg ml^−1^) when required. For plate cultures, medium was supplemented with 1.5% (wt/vol) agar.

### Synechocystis transformation

*Synechocystis* strains were grown in 50 ml of BG11C until exponential phase (OD_750nm_ = 0.5–1) and were refreshed daily, at least three time, to keep them between these OD values. Cells were centrifuged at 4,500*g* for 10 min at 25°C and washed twice with 1 volume of fresh medium. After the second wash, supernatant was discarded, and the pellet was resuspended in 1 ml of BG11C. Cell suspension of 300 µl was transferred to sterile 10-ml transparent polystyrene tubes (Soria Genlab, catalog no. T1611E), 3 µg of purified DNA was added, and cells were incubated at 30°C in the light (50 µmol photon m^−2^ s^−1^) for 3 h with occasional gentle agitation. Cells were plated on Nitrocellulose filters (IMMOBILION-NC, Millipore, catalog no. HAT08550) placed on BG11C and incubated for 24 h under low light (10 µmol photon m^−2^ s^−1^). Filters were transferred to BG11C plus the selective agent and incubated for 4–6 days in the same conditions until colonies appeared. Colonies were re-streaked at least twice in selective media before PCR analysis for insertion of the constructs and full segregation.

### Plasmid construction

For inactivation of *slr2094*, the whole ORF was amplified by PCR (using oligonucleotides 142 and 143) and cloned in pSPARKII. Then, a erythromycin resistance cassette was introduced in the *Kpn*I site internal to the gene generating pSLR2094:Ery. For *slr0952* inactivation, whole ORF was PCR-amplified (using 144 and 145 oligonucleotides) and cloned in pSPARKII, and a Sp/St cassette was introduced in the *Xba*I site that is internal to the ORF-generating pSLR0952::Sp. For AtSBPase, PsFBPase, and AtTrxF1 expression, *Xba*I–*Xho*I fragments from pET28_AtSBPase, PAMC100, and pET28_trxF1 were cloned in pNRSD_PcpcB_Km, pGLNN_PcpcB_Nat, and pARSB_PcpcB_Sp, respectively, and digested with the same enzymes. These constructs express the genes under the control of the strong constitutive *cpcB* promoter. All plasmids used in this work are described in **Table S3**.

### Protein expression and purification

PsFBPase was expressed using pAMC100 plasmid (Carrasco et al., 1994). For AtSBPase, a full-length ORF lacking the transit peptide was amplified by PCR from cDNA prepared from WT Arabidopsis seedlings using oligonucleotides 264 and 265 and cloned into pET28 digested with *Nde*I and *Xho*I generating pET28_AtSBPase. *E. coli* BL21 (DE3) carrying the corresponding plasmids were grown in LB at 37°C until they reached ~0.5 OD_600nm_, cultures were chilled to 0°C, gene expression was induced by adding 0.2 mM isopropyl‐β‐thio galactopyranoside (IPTG), and the culture was incubated at 25°C for 24 h after induction. Cells were collected by centrifugation at 6,000*g* and were frozen. Cells were suspended in 50 mM Tris-HCl (pH 8), 300 mM NaCl, 5 mM imidazole (buffer A), and 1 mM phenylmethylsulfonyl fluoride (PMSF) was added. The suspension was sonicated and centrifuged for 30 min at 20,000 *g*, and the supernatant was used as the crude extract that was applied to a 2-ml column super Ni-NTA sepharose (IBA, catalog no. 2-3201). The column was washed with 20 ml of buffer A and 40 ml of buffer A supplemented with 50 mM Imidazol, and the proteins were eluted with 10 ml of buffer A supplemented with 300 mM imidazole. Imidazol was removed by filtration using PD10 desalting columns (GE Healthcare, catalog no. 17085101) in 50 mM Tris-HCl (pH 8) and 150 mM NaCl buffer according to the manufacturer’s instructions

### Fructose-1,6-bisphosphatase activity assay

The activity was measured on the basis of the phosphate liberated from fructose-1,6-bisphosphate, and this phosphate becomes visible after reaction with a Fiske–Subbarow solution [H_2_SO_4_, 9N; (NH_4_)_6_Mo_7_O_4_•4H_2_O, 0.053M; FeSO_4_ • 7H_2_O, 0.30M], which was measured at 660_nm_. Inorganic phosphate standard including all reagents used in every assay was determined for each experiment. The assays (175 µl) contain 15 mM MgSO_4_ • 7H_2_O and 150 mM Tris-HCl (pH 8), and the indicated amounts of dithiothreitol (DTT) were incubated at room temperature for 15 min before fructose-1,6-bisphosphate (7.15 mM) was added to start the reaction. The mixture was then incubated at 30°C in a water bath for 10–30 min depending on the experiment. Finally, the reaction was stopped with 1 ml of a Fiske–Subbarow solution. For *in situ* determination using whole cells, two samples of 5 OD_750nm_ from each culture were resuspended in 293 µl of 150 mM Tris-HCl (pH 8), 15–150 mM MgSO_4_, 7 µl of a 2% (wt/vol) mixed alkyltrimethylammonium bromide solution and were vortexed for 5 s. Fructose-1,6-bisphosphate (7.15 mM) was added to one tube to start the reaction and water to the other, which will serve as a control. Reactions were incubated at 30°C for 30 min and centrifuged at 13,000*g* for 5 min to remove the cells, the supernatant was transferred to a new tube, 2 ml of a Fiske–Subbarow solution was added, and the color was allowed to develop for 15 min before measuring. The activity was calculated by the difference absorbance of the sample minus the control without substrate.

### Western blot and alkylation treatment

For soluble extracts, 20 OD_750nm_ were collected by centrifugation (at 4,000*g* for 10 min), the supernatant discarded, and pellets were frozen in liquid nitrogen. Cells were resuspended in 200 µl of buffer A [50 mM Tris-HCl (pH 8) and 50 mM NaCl buffer] and broken using 500 µl of 0.2- to 0.3-mm glass beads using a mini-bead beater. Cells were subjected to two cycles of 1 min of vortexing separated by 5 min on ice. Cell extracts were recovered from the beads, and samples were clarified by two sequential centrifugations: 5 min at 5,000*g* to remove cell debris and 15 min at 15,000*g* to remove membranes. The protein concentration in cell-free extracts or purified protein preparations was determined using the Bradford’s method, using ovalbumin as standard, and the specified amounts of proteins were separated on SDS-PAGE under reducing or non-reducing conditions. Gels were transferred, blocked in PBS containing 0.1% Tween 20 and 5% skimmed milk, and incubated with indicated antibodies 1:20,000 (anti-SBP/FBPase, anti-FBPase, anti-PsFBPase, and anti-TrxF antibodies), except for anti-His, which was used at 1:1,000 dilution. Horseradish peroxidase–conjugated secondary antibody (1:25,000; Sigma, catalog no. A6154) and Clarity™ Western ECL Substrate (Bio-Rad, catalog no. 1705060) were used to detect chemiluminescent signal by ImageQuant 800 imaging systems (Amersham) or X-ray films.

For alkylation assays, cells (20 OD_750nm_) were treated with 10 mM iodoacetamide (IAA; Sigma, catalog no. I1149) for 15 min on ice, collected by centrifugation (at 4000*g* for 10 min), washed with 1 ml of buffer A buffer containing 10 mM N-ethylmaleimide (NEM), and were frozen. After this, cells were resuspended in 200 µl of 1 mM PMSF, 1 mM EDTA, 50 mM Tris-HCl (pH 8), 50 mM NaCl, and 10 mM NEN buffer and broken as previously. The soluble extracts were divided into three equal aliquots, precipitated with 10% trichloroacetic acid (TCA), and washed twice with TCA acetone. An aliquot was used as the control, and the other two were reduced with 100 mM DTT in 100 µl of buffer B (buffer A containing 2% SDS) precipitated with TCA and acetone again. Of these two samples, one was resuspended directly in buffer B and used as the reduced control, and the other was resuspended in buffer B supplemented with 10 mM methyl-PEG24-maleimide [MM(PEG)24] and incubated at 37°C for 30 min. All three samples were resuspended in 50 µl of buffer A containing 1% SDS. Proteins were separated in 12% SDS-PAGE under non-reducing conditions.

### Oxygen evolution

Oxygen evolution was measured in by a Clark-type oxygen electrode (Hansatech Chlorolab 2) using mid-logarithmic (OD_750nm_ = 0.8–1) cultures adjusted to OD_750nm_ = 0.5 in BG11C, in BG11C supplemented with 10 mM glucose, or in BG11C with 10 mM glucose and 20 µM DCMU at 30°C supplemented with 20 mM NaHCO_3_ using white LED light. For respiration assays cells, were resuspended in fresh media and incubated 5 min in the dark, 15 min at growth light, and 1 h in the dark. The oxygen consumption rate of this latter 1-h dark period is the reported respiration rate.

### Glucose determination and glycogen determination

Glucose was determined using the glucose oxidase-peroxidase enzymatic assay using a commercial kit (Sigma, catalog no. GAGO20) using a glucose standard prepared in BG11C to calibrate it. Glycogen content was determined as described in (Díaz-Troya et al., 2014).

## Results

### SBPase/FBPase is essential for photoautotrophic growth

**Figure 1:**
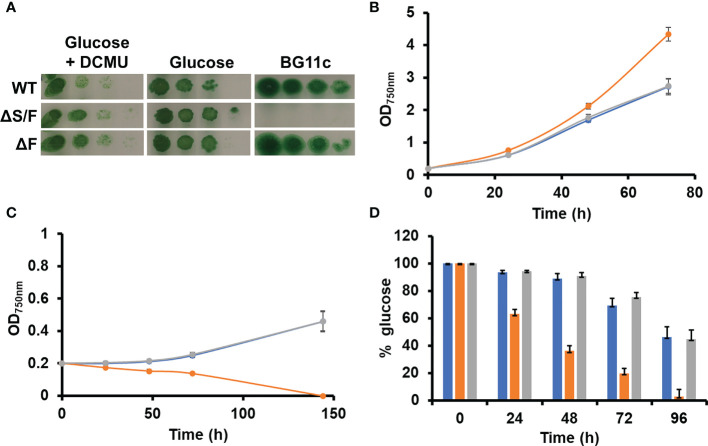
Growth of WT, ΔS/F, and ΔF strains in different trophic regimes. **(A)** Growth of WT, ΔS/F, and ΔF in different trophic regimes in solid media. Tenfold serial dilutions of a suspension of 1 µg of chlorophyll ml^−1^ cells grown in BG11C supplemented with glucose were spotted onto plates of BG11C supplemented with 10 mM glucose and 20 µM DCMU, BG11C supplemented with 10 mM glucose, or BG11C. Plates were incubated at 5 µmol photon m^−2^ s^−1^ and photographed after 10 days of growth. **(B)** Photomixotrophic growth of WT, ΔS/F, and ΔF in liquid media. WT (○), ΔS/F (○), and ΔF (○) were grown in BG11C + 10 mM glucose in low light until the exponential phase. Cells were washed twice in BG11C and inoculated in BG11C supplemented with 10 mM glucose at OD_750nm_ 0.2 under the same conditions. Growth was monitored by measuring OD_750nm_. Data represented are the mean and standard error of three to four (depending on the time point) biological independent cultures. **(C)** Photoautotrophic growth of WT, ΔS/F, and ΔF in liquid media. WT (○), ΔS/F (○), and ΔF (○) were grown in BG11C in low light until the exponential phase. Cells were washed twice in BG11C and inoculated in BG11C OD_750nm_ 0.2 under the same conditions. Growth was monitored by measuring OD_750nm_. Data represented are the mean and standard error of three to four (depending on the time point) biological independent cultures. **(D)** Glucose consumption in WT, ΔS/F, and ΔF. WT (○), ΔS/F (○), and ΔF (○) were grown in BG11C + 10 mM glucose in low light until the exponential phase. Cells were washed twice in BG11C and inoculated in BG11C supplemented with 10 mM glucose. Glucose present in the media was determined as described, and the % of the total glucose present is represented. Data are the mean ± SE of at least three biologically independent experiments.

**Figure 2:**
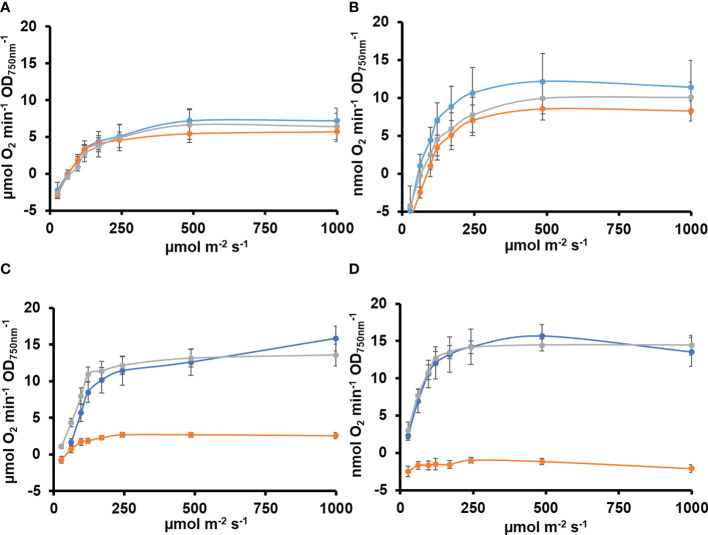
Oxygen evolution in WT, ΔS/F, and ΔF strains in different trophic regimes. **(A)** Oxygen evolution measured using a Clark electrode at increasing light intensities in exponential growing cultures (OD_750nm_= 0.5–1) of WT (○), ΔS/F (○), and ΔF (○) grown in BG11C + 10 mM glucose for 24 h. Data are the mean ± SE of at least three biologically independent experiments. **(B)** Oxygen evolution measured using a Clark electrode at increasing light intensities in exponential growing cultures (OD_750nm_= 0.5–1) of WT (○), ΔS/F (○), and ΔF (○) grown in BG11C + 10 mM glucose for 48 h. Data are the mean ± SE of at least three biologically independent experiments. **(C)** Oxygen evolution measured using a Clark electrode at increasing light intensities in exponential growing cultures (OD_750nm_= 0.5–1) of WT (○), ΔS/F (○), and ΔF (○) grown in BG11C + 10 mM glucose and shifted to BG11C for 24 h. Data are the mean ± SE of at least three biologically independent experiments. **(D)** Oxygen evolution measured using a Clark electrode at increasing light intensities in exponential growing cultures (OD_750nm_= 0.5–1) of WT (○), ΔS/F (○), and ΔF (○) grown in BG11C + 10 mM glucose and shifted to BG11C for 48 h. Data are the mean ± SE of at least three biologically independent experiments.

All cyanobacteria code for a single protein is able to catalyze two essential activities in the CBB cycle FBPase (EC 3.1.3.11) and SBPase (EC 3.1.3.37). Most genomes include an additional gene coding for a protein with only FBPase activity. In *Synechocystis*, the *slr2094* gene codes for a bifunctional enzyme, whereas *slr0952* codes for an enzyme with only FBPase activity. Individual gene inactivation of both genes was possible in *Synechocystis-*generating ΔS/F (slr2094::Ery) and ΔF (slr0952::Sp) strains. The ΔS/F strain was only possible to obtain as a fully segregated mutant when cultured in the presence of glucose and was unable to grow in its absence (**Figures 1A** and **S1**), as previously described (Yan and Xu, 2008). A double mutant lacking both genes was not viable as we could not inactivate *slr2094* in the ΔF strain (**Figure S1**). Unexpectedly, the ΔS/F strain grew faster than WT and ΔF strains in glucose supplemented media (**Figure 1B**) but was unable to grow and died within 48 h when transferred to glucose-free media (**Figure 1C**). In addition, the ΔS/F strain consumed the glucose present in the media faster (**Figure 1D**) and showed a higher respiration rate (4.2 ± 0.3 µmol min^−1^/OD_750nm_) than the WT (3.26 ± 0.3 µmol min^−1^/OD_750nm_) or ΔF strain (3.6 ± 0.6 µmol min^−1^/OD_750nm_). Oxygen evolution was also analyzed under these conditions. The ΔS/F strain showed slightly lower oxygen evolution at higher light intensities (and saturates at lower light intensities; **Figures 2A, B**) when cultured in the presence of glucose but showed residual or negative oxygen evolution after glucose removal (**Figures 2C, D**). This suggests that, in the absence of glucose, it is not possible to re-generate CBB and oxidative pentose pathway intermediates, leading to a metabolic blockage. Therefore, electrons from the photosynthetic reactions could not be used, causing the blockage of the photosynthetic electron transfer chain.

### Plant SBPase is not sufficient to complement an SBP/FBPase mutant strain

**Figure 3:**
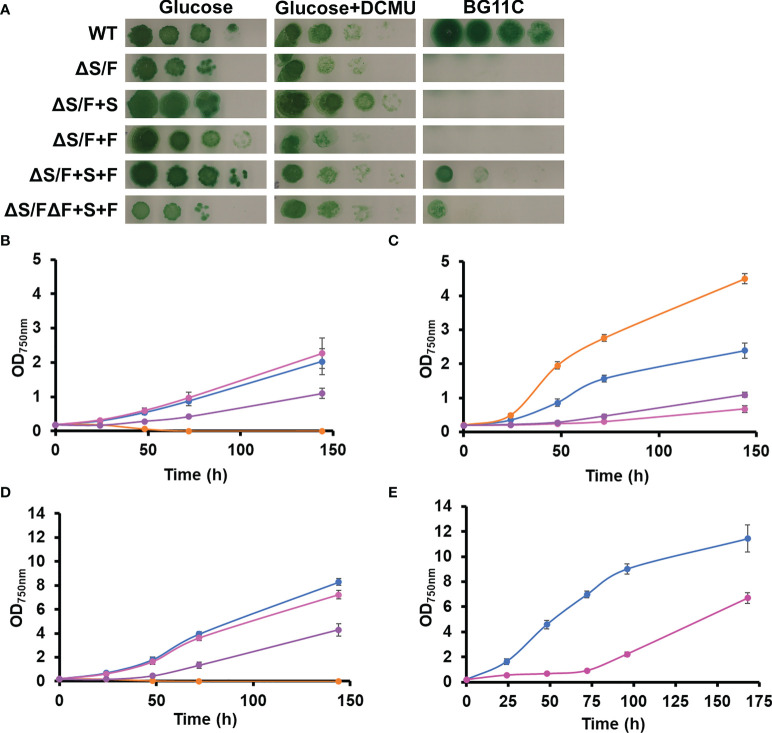
Complementation of ΔS/F mutant strain with FBPase and SBPase plant genes. **(A)** Growth of WT, ΔS/F, ΔS/F+S, ΔS/F+F, ΔS/F+S+F, and ΔS/FΔF+S+F in different trophic regimes. Cultures were grown in BG11C supplemented with 10 mM glucose light until the exponential phase washed twice with BG11C, and 10-fold serial dilutions of a suspension of 1 µg chlorophyll ml^−1^ cells were spotted onto BG11C, BG11C supplemented with 10 mM glucose, or BG11C supplemented with 10 mM glucose and 20 µM DCMU. Plates were incubated at 5 µmol photon m^−2^ s^−1^ and photographed after 10 days of growth. **(B)** Photomixotrophic growth of WT, ΔS/F, ΔS/F+S, ΔS/F+F, ΔS/F+S+F, and ΔS/FΔF+S+F in liquid media. WT (○), ΔS/F (○), ΔS/F+S+F (○), and ΔS/FΔF+S+F (○) were grown in BG11C + 10 mM glucose in low light until the exponential phase. Cells were washed twice in BG11C and inoculated in BG11C supplemented with 10 mM glucose at OD_750nm_ 0.2 bubbled with 1% CO_2_-enriched air at 5 µmol photon m^−2^ s^−1^. Growth was monitored by measuring OD_750nm_. Data represented are the mean and standard error of three to four (depending on the time point) biological independent cultures. **(C)** Photoautotrophic growth of WT, ΔS/F, ΔS/F+S, ΔS/F+F, ΔS/F+S+F, and ΔS/FΔF+S+F in liquid media. WT (○), ΔS/F (○), ΔS/F+S+F (○), and ΔS/FΔF+S+F (○) were grown in BG11C in low light until the exponential phase. Cells were washed twice in BG11C and inoculated in BG11C at OD_750nm_ 0.2 bubbled with 1% CO_2_-enriched air at 5 µmol photon m^−2^ s^−1^. Data represented are the mean and standard error of three to four (depending on the time point) biological independent cultures. **(D)** Photoautotrophic growth of WT, ΔS/F, ΔS/F+S, ΔS/F+F, ΔS/F+S+F, and ΔS/FΔF+S+F in liquid media. Conditions were the same as in **(C)** except for the light that was 50 µmol photon m^−2^ s^−1^. Data represented are the mean and standard error of three to four (depending on the time point) biological independent cultures. **(E)** Photoautotrophic growth of WT and ΔS/F+S+F in liquid media. WT (○) and ΔS/F+S+F (○) were grown in BG11C in low light until the exponential phase. Cells were washed twice in BG11C and inoculated in BG11C glucose at OD_750nm_ 0.2, bubbled with 1% CO_2_-enriched air at 185 µmol photon m^−2^ s^−1^. Data represented are the mean and standard error of three to four (depending on the time point) biological independent cultures.

Because SBP/FBPase can perform two activities, we wanted to analyze which of these two activities was the essential one in *Synechocystis*. For that, a gene for a plant FBPase (*Pisum sativum* FBPase; Carrasco et al., 1994) and a plant SBPase (Arabidopsis SBPase; At3G55800, AtSBPase) were cloned under the control of a strong constitutive *cpcB* promoter in plasmids, targeting the neutral sites *glnN* (Reyes and Florencio, 1994) and *nrsD* (Garcia-Dominguez et al., 2000), respectively (**Table S2**). These plasmids were transformed in a WT background generating the +F and +S strains (**Table S1**), and, subsequently, these strains were transformed with pSLR2094:Ery plasmid to inactivate *slr2094*, generating the ΔS/F+F and ΔS/F+S strains (**Table S1**, **Figures S1–S3**). Considering that *Synechocystis* contains an additional enzyme with FBPase activity (*slr0952*), the prediction was that only the SBPase activity was needed and, therefore, the ΔS/F+S strain should be able to grow photoautotrophically. Unexpectedly, introduction of the plant genes did not complement the ΔS/F strain as none of the generated strains (the ΔS/F+F and ΔS/F+S strains) were able to grow photoautotrophically but both segregated and grew in the presence of glucose (**Figure 3**). However, introduction of any of the plant genes allowed the segregation of a double mutant with both the SBP/FBPase (*slr2094*) and FBPase (*slr0952*) endogenous genes inactivated (generating the ΔS/FΔF+S and ΔS/FΔF+F strains), which does not segregate in a WT background (**Figure S1**). Next, we generated strains containing both plant genes in a WT or ΔF backgrounds (+S+F and ΔF+S+F strains, respectively) and transformed with pSLR2094:Ery as before. In this case, inactivation of *slr2094* was possible in the absence of glucose for both backgrounds, and the new generated strains (ΔS/F+S+F and ΔS/FΔF+S+F) were able to grow photoautotrophically (**Figures 3A, B**). Nevertheless, these strains showed impaired growth even at low light intensities (5 µmol m^−2^ s^−1^) with the ΔS/FΔF+S+F strain being much more affected than the ΔS/F+S+F strain (**Figure 3C**). The ΔS/FΔF+S+F was unable to grow at 50 µmol m^−2^ s^−1^ (**Figure 3D**), and the ΔS/F+S+F strain showed a very long lag phase (72 h) when grown at 185 µmol m^−2^ s^−1^, and although, it resumed growth after the lag phase, it was slower than the WT strain (**Figure 3E**).

### Plant FBPase is redox-regulated by light in cyanobacteria

**Figure 4:**
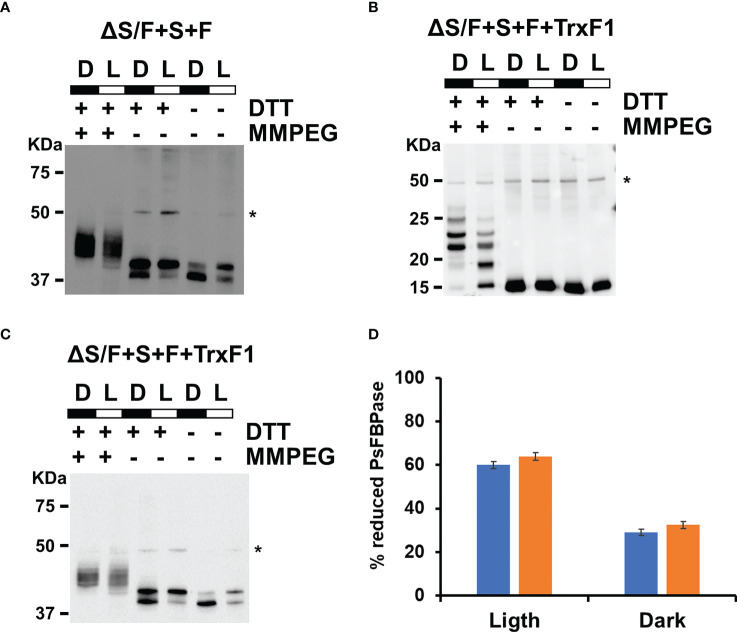
Both PsFBPase and TrxF1 are light regulated in *Synechocystis*. **(A)** *In vivo* PsFBPase L/D regulation analyzed by Western blot in the ΔS/F+S+F strain. Proteins were extracted from labeling of ΔS/F+S+F cells in the light (L) and after 24 h in the dark **(D)** and labeled with the alkylating agent MM(PEG24) and analyzed using anti-PsFBPase as described in the “*Materials and methods*”. * Denotes a cross reacting band. **(B)** *In vivo* TrxF1 L/D regulation analyzed by Western blot in the ΔS/F+S+F+TrxF strain. Proteins were extracted from labeling of ΔS/F+S+F+TrxF cells in the light (L) and after 24 h in the dark **(D)** and labeled with the alkylating agent MM(PEG24) and analyzed using anti-TrxF1 antibodies as described in the “*Materials and methods*”. * Denotes a cross reacting band. **(C)** Western blot L/D regulation *in vivo* of PsFBPase in the ΔS/F+S+F+TrxF strain. Proteins were extracted from labeling ΔS/F+S+F+TrxF cells in the light (L) and after 24 h in the dark **(D)** and labeled with the alkylating agent MM(PEG24) and analyzed using anti-PsFBPase as described in the “*Materials and methods*”. * Denotes a cross reacting band. **(D)** Quantification of the reduced and oxidized forms of PsFBPase in ΔS/F+S+F (blue bars) and ΔS/F+S+F+TrxF strains (orange bars) in the light and in the dark.

Plant CBB is finely tuned to adapt to changing light conditions, and, for that, several enzymes of the cycle are regulated by multiple mechanisms (Cejudo et al., 2019). One of the best studied ones is the redox regulation of the enzymes. FBPase and SBPase are the two of the best studied enzymes in the cycle. These two proteins are active in their reduced form, and the FTR-Trx system is responsible for its reduction in the light (Buchanan, 2016; Gütle et al., 2016; Naranjo et al., 2016; Geigenberger et al., 2017; Ojeda et al., 2017; Yokochi et al., 2019). As plant SBPase and FBPase are from a different origin evolutionary to cyanobacterial SBP/FBPase and FBPase (Gütle et al., 2016), we wanted to test whether these plant enzymes were regulated by the cyanobacterial Trx system. For this, we analyzed *in vivo* the redox state of the plant FBPase using the alkylating agents NEM and MM(PEG)24, and the protein was detected by Western blot analysis. Extracts were prepared from cells of the ΔS/F+S+F strain that were grown under our standard light conditions (50 µmol m^−2^ s^−1^) or that were maintained in the dark for 24 h. As shown in **Figure 4A** for PsFBPase, two bands are detected in NEM-treated extracts, and several shifted bands, which were not well resolved and which correspond to different oxidized forms, could be detected after MM(PEG)_24_ labeling. Quantification of the reduced and oxidized form after NEM treatment showed that PsFBPase is more reduced (59.9%) in the light than that in the dark (28.9%). This result shows that the cyanobacterial redox system, most probably thought TrxA (*m*-type) as the main thioredoxin in *Synechocystis* (Mallén-Ponce et al., 2021), can reduce PsFBPase in a light-dependent manner even if it has a different evolutionary origin.

### TrxF improves complementation of SBP/FBPase mutant strains

**Figure 5:**
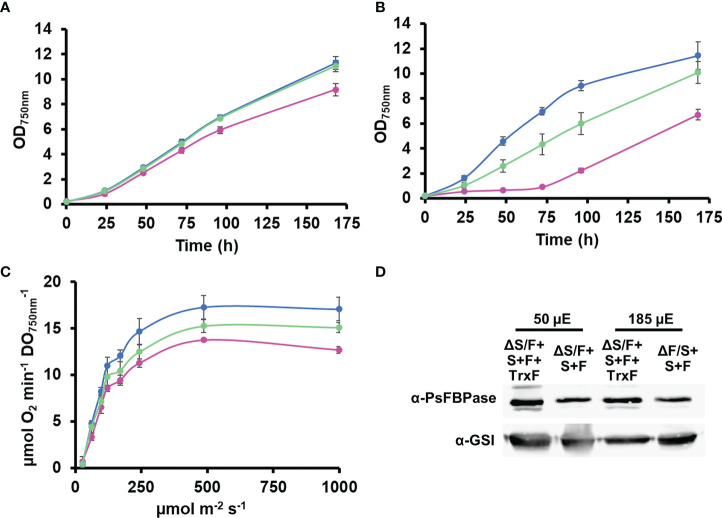
Expression of TrxF improves ΔS/FΔF+S+F growth. **(A)** Photoautotrophic growth of WT and ΔS/F+S+F in liquid media. WT (○), ΔS/F+S+F (○), and ΔS/F+S+F+TrxF (○) were grown in BG11C in low light until the exponential phase. Cells were inoculated in BG11C glucose at OD_750nm_ 0.2 and bubbled with 1% CO_2_-enriched air at 50 µmol photon m^−2^ s^−1^. Data represented are the mean and standard error of three to four (depending on the time point) biological independent cultures. **(B)** Photoautotrophic growth of WT and ΔS/F+S+F in liquid media. WT (○), ΔS/F+S+F (○), and ΔS/F+S+F+TrxF (○) were grown in BG11C in low light until the exponential phase. Cells were inoculated in BG11C at OD_750nm_ 0.2 and bubbled with 1% CO_2_-enriched air at 185 µmol photon m^−2^ s^−1^. Data represented are the mean and standard error of three to four (depending on the time point) biological independent cultures. **(C)** Oxygen evolution measured using a Clark electrode at increasing light intensities in exponential growing cultures (OD_750nm_= 0.5-1) of WT(○), ΔS/F+S+F (○), and ΔS/F+S+F+TrxF (○) grown in BG11C bubbled with 1% CO_2_-enriched air at 50 µmol photon m^−2^ s^−1^. Data are the mean ± SE of at least three biologically independent experiments. **(D)** Immunoblot analysis of PsFBPase protein in ΔS/F+S+F and ΔS/F+S+F+TrxF strains. Cells were grown in BG11C bubbled with 1% CO_2_-enriched air at 50 or 185 µmol photon m^−2^ s^−1^. Ten micrograms of total protein from soluble extracts were separated by 12% SDS-PAGE and subjected to Western blot to detect FBPase and GSI as loading control.

As both SBPase and FBPase has been shown to be reduced more efficiently by Trx *f* thioredoxins in plant chloroplast (Yoshida and Hisabori, 2016; Ojeda et al., 2017; Yokochi et al., 2019) than by Trx *m*, we decided to introduce a gene coding for a Trx *f*, which is not present in cyanobacteria. For that, we introduced a cDNA version, the TRXF1 gene from Arabidopsis, lacking the transit peptide, into the +S+F strain, generating +S+F+TrxF strain, and then, *slr2094* was inactivated in this background, generating the ΔS/F+S+F+TrxF strain (**Figure S5**). This new strain showed improved growth when compared to the ΔS/F+S+F, being indistinguishable from the WT strain at 50 µmol m^−2^ s^−1^ (**Figure 5A**) and growing faster than the ΔS/F+S+F strain, and without a lag phase, at 185 µmol m^−2^ s^−1^ (**Figure 5B**). Oxygen evolution showed a similar effect with the ΔS/F+S+F+TrxF strain performing better than the ΔS/F+S+F strain but slightly worse than the WT strain (**Figure 5C**). As the expression of TrxF1 improved growth and photosynthetic performance, we wanted to analyze if there were any changes in expression levels of the plant enzymes. For that, we determined PsFBPase expression by Western blot and FBPase activity in whole cells. PsFBPase expression levels were higher in strains expressing TrxF1 than in the control strain lacking it both at 50 and 185 µmol m^−2^ s^−1^ (**Figure 5D**). Furthermore, total FBPase activity (which is the sum of all SBPase and FBPase present in the different strains) was also higher in the ΔS/F+S+F+TrxF strain than in ΔS/F+S+F strain at both light intensities (**Figure S6**). All strains expressing plant proteins showed a higher Mg dependency in the *in situ* assays with their activities increasing up to 150 mM Mg, whereas the WT strain was already saturated at this concentration (**Figure S6**). All these data suggest that co-expression of the plant TrxF1 improves either stability or folding of the proteins. Finally, we have analyzed the redox status of both TrxF and PsFBPase in the ΔS/F+S+F+TrxF strain using the alkylation protocol. Four bands could be detected for TrxF1, which correspond to completely reduced protein (the faster migrating band) or one, two, or three oxidized cysteines (slower migrating bands). TrxF1 is almost completely oxidized in the dark and partially reduced in the light (**Figure 4B**), which shows that *Synechocystis*’ FTR is able to reduce Arabidopsis TrxF1 and that the redox status of the protein is coupled to light availability in a similar way to plant chloroplast. When we analyzed the redox state of PsFBPase, it showed that the percentage of reduced protein slightly increases in a strain expressing TrxF1 compared to the strain lacking it both in the light and in the dark (**Figures 4C, D**). All these data shows that the cyanobacterial redox system can reduce the TrxF-FBPase (and probably also SBPase) redox axis despite these proteins not being present in cyanobacteria and having a complete different evolutionary origin.

## Discussion

### Why FBPase activity is needed for complementation

The dual SBP/FBPase enzyme is essential for photoautotrophic growth in cyanobacteria (**Figure 1**; Yan and Xu, 2008) as expected by the lack of a functional CBB cycle because this enzyme presents two of the irreversible reactions of the cycle. Most cyanobacterial genomes code for an additional enzyme that shows FBPase activity; therefore, the only activity missing in mutants lacking the SBP/FBPase is SBPase. Introduction of a plant SBPase gene in the ΔS/F mutant was expected to complement this strain although this was not enough to restore photoautotrophic growth. This suggests that the flux achieved by the native FBPase could not support an active CBB cycle and that might be the reason of this lack of complementation. This could be due to low levels of expression or activity reached by the plant SBPase introduced, different regulation of the native FBPase activity, or a combination of both. The fact that it is possible to obtain a double mutant lacking both SBP/FBPase and FBPase genes when either plant SBPase or FBPase genes are introduced individually into *Synechocystis* is probably due to need of FBPase activity to use glucose as carbon source when CBB is not operative. This can be provided by either the plant FBPase or the residual FBPase activity that the plant SBPase presents (Gütle et al., 2016). Therefore, it is possible that, under the conditions used here, the OPP is essential for growth using glucose, despite earlier claims that Entner–Doudoroff pathway is the main catabolic route for glucose utilization in the light (Chen et al., 2016; Makowka et al., 2020). It is also possible that inactivation of SBP/FBPase gene (*slr2094*) generates a metabolic state that requires the use of the OPP to regenerate some of the metabolites of the central carbon metabolism (Sharkey and Weise, 2016; Makowka et al., 2020), preventing metabolic blockage. The ΔS/F strain dies after glucose removal and is not able to perform photosynthesis after 24 h, despite that it is consuming its glycogen and, therefore, it has an internal source of glucose (**Figure S7**). On the other hand, the ΔS/F strain showed faster growth in the presence of glucose in the light than the WT strain (and the ΔF strain), suggesting a more efficient glucose utilization. This could be due to the lack of futile cycles between CBB, OPP, and glycolysis (Sharkey and Weise, 2016) or accumulation of other metabolites that could improve other glucose-utilizing routes (Chen et al., 2016; Makowka et al., 2020; Lucius et al., 2021). Whether the ΔS/F strain is able to fix CO_2_ in the light (both in the presence or absence of glucose) is not known.

Only the introduction of both FBPase and SBPase genes in *Synechocystis* allowed growth of a ΔS/F mutant strain in the absence of glucose, although at reduced rates and only under low light (5 µmol photons m^−2^ s^−1^). This strain was extremely sensitive to light, which might be related to either low activity of the plant proteins or incorrect regulation of its activity. *In situ* analysis of FBPase activity of these strains showed that the strains expressing the plant enzymes required higher Mg concentrations for maximal activity. This agrees with higher Mg dependency of plant chloroplast enzymes and pointing to a suboptimal activity of these enzymes (**Figure S6**). An additional level of regulation is mediated by enzyme reduction, which is mediated by thioredoxins. *In vitro* Trx *f* is the most efficient reductant for both FBPase and SBPase (Yoshida and Hisabori, 2016; Ojeda et al., 2017; Yokochi et al., 2019), although *in vivo* both Trx *f* and Trx *m* seem to be effective (Naranjo et al., 2016; Yoshida and Hisabori, 2016; Okegawa et al., 2022). Trx *f* is essential for short-term light activation and Trx *m* for higher levels of reduction at longer time points despite the *in vitro* preference for Trx *f* (Okegawa et al., 2022). In addition, these strains expressing plant genes showed reduced growth when compared to the ΔS/F strain in the presence of glucose (**Figures 3C**, **S4**), which suggest that a balanced activity of FPBase and SBPase activities is required for optimized fitness under all conditions.

In *Synechocystis*, PsFBPase is partially reduced (**Figure 4A**), and this reduction is diminished in the dark, suggesting that is light regulated. This reduction is most likely mediated by TrxA, the *m*-type thioredoxin in cyanobacteria, showing that, as in plant chloroplast, *m*-type thioredoxins are able to reduce FBPase *in vivo* at least partially (Yoshida and Hisabori, 2016). When TrxF1 was expressed in this background, the growth and the total FBPase activity increase, showing that TrxF1 co-expression has a beneficial effect on the activity of these plant proteins (**Figures 5**, **S6**). Nevertheless, the ΔS/F+S+F+TrxF was not able to grow like the WT in moderate light conditions (185 µmol photons m^−2^ s^−1^), suggesting that regulation of the activity in coordinated manner with the rest of the CBB is essential for optimal fitness. Improvement of enzyme activity can be mediated by higher reduction or increased levels of the protein. Although, the ratio of reduced PsFBPase slightly increases in the strain in which TrxF1 is expressed (**Figure 4**) and increase in total protein levels is most likely the responsible for this improvement. This suggests that Trx *f* could have a chaperone function as it has been suggested before (Sanz-Barrio et al., 2012; Du et al., 2015). Our results also show that, despite the plant proteins introduced in this work are not present in *Synechocystis* (or any other cyanobacteria) and have a different evolutionary origin in all three cases (Sahrawy et al., 1996; Capitani et al., 2000; Gütle et al., 2016; Mallén-Ponce et al., 2022), it can be reduced by the cyanobacterial FTR-Trx system. These results suggest that, when these proteins were acquired by the primitive photosynthetic eukaryote, they could be reduced by the chloroplast redox–reducing system.

One main difference between redox regulation in plant chloroplast and cyanobacteria is the lack of complete oxidation of Trx target proteins in the dark (**Figure 4**; Mallén-Ponce et al., 2021). This can be related to the different physical organization of the cyanobacteria, in which there is no physical separation between photosynthetic and respiratory metabolism, whereas in plant cells, these two processes are carried out in different organelles. In addition, in recent years, it has been described that thioredoxin target proteins are oxidized in the dark by Trx-like proteins and Trxs themselves, which ultimately donate their electrons to H_2_O_2_ though NTRC and 2cys-prx (Balsera and Buchanan, 2019; Yoshida et al., 2019; Yokochi et al., 2021). Although NTRC and 2cys-prx are present in many cyanobacteria, NTRC is not present in *Synechocystis* (Mallén-Ponce et al., 2022), and the 2cys-prx from *Synechocystis* presents different biochemical characteristics to the plant enzyme (Pascual et al., 2010; Pascual et al., 2011). Cyanobacteria also lack Trx-like proteins that are essential for oxidation in the dark, and, therefore, this might explain why thioredoxin targets are less oxidized in cyanobacteria. Furthermore, cyanobacterial Trx is not oxidized in the dark to the same extent as to plant Trx (Mallén-Ponce et al., 2021).

Finally, our system will allow to analyze the specificity of the redox regulation of the different plant proteins, including the different isoforms of the same protein or site directed mutants, by an *in vivo* setting close to plant chloroplast. This approach if used in other strains containing NTRC, such as *Anabaena* sp. PCC 7120 (Sánchez-Riego et al., 2016), could also allow to analyze its role in redox regulation. It also opens the possibility to study whether Trx *f* could substitute Trx *m* in cyanobacteria as they have overlapping function in redox regulation in plant chloroplast *in vivo* (Naranjo et al., 2016; Yoshida and Hisabori, 2016; Ojeda et al., 2017; Okegawa et al., 2022).

## Data availability statement

The original contributions presented in the study are included in the article/**Supplementary Material**. Further inquiries can be directed to the corresponding authors.

## Author contributions

RG-C and LL-M performed experiments. RG-C, FF and LL-M analyzed the data and wrote the manuscript. LL-M and FF designed and supervised the overall study. All authors contributed to the article and approved the submitted version.

## Funding

This work was supported by grant PID2019-104513GB-I00 funded by MCIN/AEI/10.13039/501100011033 to FJF.

## Acknowledgments

Maryam Sahrawy is acknowledged for kindly providing pAMC100 and anti PsFBPase antibodies. Juan M. Pérez-Ruiz is acknowledged for providing pET28-trxF1 and anti-TrxF antibodies. Maria J. Huertas and Irene Tejada are acknowledged for construction of pSLR0952:Sp and pARSB_PcpcB_Sp, respectively.

## Conflict of interest

The authors declare that the research was conducted in the absence of any commercial or financial relationships that could be construed as a potential conflict of interest.

## Publisher’s note

All claims expressed in this article are solely those of the authors and do not necessarily represent those of their affiliated organizations, or those of the publisher, the editors and the reviewers. Any product that may be evaluated in this article, or claim that may be made by its manufacturer, is not guaranteed or endorsed by the publisher.
