# Supplementary material for: Back to the future: Transplanting the chloroplast TrxF–FBPase–SBPase redox system to cyanobacteria
Source: Front Plant Sci. 2022 Nov 28;13:1052019. doi: 10.3389/fpls.2022.1052019 (PMC9742560; doi:10.3389/fpls.2022.1052019)
Supplement: Supplementary file 1 [file DataSheet_1.docx]

Table S1. Strains used in this work.

| **Strain** | **Genotype** | **Mutated ORF** |
| --- | --- | --- |
| WT | *Synechocystis sp. PCC 6803* | *-* |
| ΔF | *slr0952*::Sp/St^R^ | *slr0952* |
| ΔS/F | *slr2094*::Ery^R^ | *slr2094* |
| +S | *nrsD*::P_cpcB_: _6His_AtSBPase:*C.K1* | *slr0796* |
| +F | *glnN*::P_cpcB_: _6His_PsFBPase:Nat^R^ | *slr0288* |
| ΔS/F +S | *nrsD*::P_cpcB_: _6His_AtSBPase:*C.K1*  *slr2094*::Ery^R^ | *slr0796*  *slr2094* |
| ΔS/F +F | *glnN*::P_cpcB_:_6His_PsFBPase:Nat^R^ *slr2094*::Ery^R^ | *slr0288*  *slr2094* |
| +S+F | *nrsD*::P_cpcB_: _6His_AtSBPase:*C.K1*  *glnN*::P_cpcB_: _6His_PsFBPase:Nat^R^: | *slr0796*  *slr0288* |
| ΔF+S | *nrsD*::P_cpcB_: _6His_AtSBPase:*C.K1*  *slr0952*::Sp/St^R^ | *slr0796*  *slr0952* |
| ΔF+F | *glnN*::P_cpcB_: _6His_PsFBPase:Nat^R^  *slr0952*::Sp/St^R^ | *slr0288*  *slr0952* |
| ΔS/F+S+F | *nrsD*::P_cpcB_: _6His_AtSBPase: *C.K1*  *glnN*::P_cpcB_:_6His_PsFBPase:Nat^R^ *slr2094*::Ery^R^ | *slr0796*  *slr2094*  *slr0288* |
| ΔF+S+F | *glnN*::P_cpcB_:_6His_PsFBPase:Nat^R^ *nrsD*::P_cpcB_: _6His_AtSBPasa:*C.K1*  *slr0952*::Sp/St^R^ | *slr0796*  *slr0952*  *slr0288* |
| ΔS/F ΔF+S | *nrsD*::P_cpcB_: _6His__AtSBPase:*C.K1*  *slr0952*::Sp/St^R^  *slr2094*::Ery^R^ | *slr0796*  *slr0952*  *slr2094* |
| ΔS/F ΔF+F | *glnN*::P_cpcB_: _6His__PsFBPase:Nat^R^  *slr0952*::Sp/St^R^  *slr2094*::Ery^R^ | *slr*0288  *slr0952*  *slr2094* |
| ΔS/F ΔF+S+F | *nrsD*::P_cpcB_: _6His_AtSBPase:*C.K1*  *glnN*::P_cpcB_:_6His_PsFBPase:Nat^R^ *slr0952*::Sp/St^R^  *slr2094*::Ery^R^ | slr0288  *slr0796*  *slr0952*  *slr2094* |
| TrxF | *arsBHC*::P_cpcB_:trxF1:Sp/St^R^ | *slr0944,*  *slr0945*  *slr0946* |
| S+F+ TrxF | *arsBHC*::P_cpcB_: _6His_trxF1:Sp/St^R^  *nrsD*::P_cpcB_: _6His_AtSBPase:*C.K1*  *glnN*::P_cpcB_: _6His_PsFBPase:Nat^R^ | *slr0944,*  *slr0945*  *slr0946*  *slr0796*  *slr0288* |
| ΔS/F +S+F+TrxF | *arsBHC*::P_cpcB_: _6His_trxF1:Sp/St^R^  *nrsD*::P_cpcB_: _6His_AtSBPase:*C.K1*  *glnN*::P_cpcB_:_6His_PsFBPase:Nat^R^ *slr2094*::Ery^R^ | *slr0944, slr0945 slr0946*  *slr0796*  *slr2094*  *slr0288* |
| ΔS/F ΔF | *slr0952*::Sp/St^R^  *slr2094*::Ery^R^ | *slr0952*  *slr2094* |

Table S2. Oligonucleotides used in this work.

| **Oligonucleotide number** | **Name** | **Sequence** |
| --- | --- | --- |
| 264 | AtSBPasa_R_XhoI | agtcctcgagCTAAGCGGTAACTCCAATGG |
| 265 | AtSBPasa_madura_F_NdeI | agtccatatgACAAAAGCTAAGAGCAATGG |
| 144 | slr0952_F_NdeI | ttttacatatgaccgttagtgagattc |
| 145 | slr0952_R_NotI | tttgcggccgctaagtaaactaattaccc |
| 142 | slr2094_F_NdeI | ttcaacatatggacagcaccctcgg |
| 143 | slr2094_R_NotI | atagcggccgcgagggaaattaatg |
| 164 | slr2094_OE_SalI | ccctctggtcgacttttgactatt |
| 204 | 5_nrsD_BssHII_F | aagcgcgcCTTTCACTGCTTGCGGAACC |
| 207 | 3_nrsD_BssHII_R | aagcgcgcCTCGGAGTCATGGTGATGGG |
| 50 | glnN_check_F | ATGCAGGCCAGTCTTCCTAA |
| 51 | glnN_check_R | AAATGGCAGTGTCCAAGTCC |
| 347 | 5_arsB_BssHI | aagcgcgcCGCCCCATCTTTAACACTTG |
| 346 | arsB_ORF_rev_XhoI_HIII | aactcgagaagcttGCTGGCAATTAGGCAAAGTT |
| 214 | Sp_R_HindIII | aAAGCTTAAGGCCATCCGTCAGGATGGCCTTCT tgcggatgttgcgattacttc |

Table S3. *Plasmids* used in this work

| Plasmid | Antibiotic resistance | Description | Source |
| --- | --- | --- | --- |
| pNRSD_PcpcB_hisAtSBPsa | Ap Km | Plasmid expressing AtSBPase gene under the control of P_cpcB_. A *Xba*I- *Xho*I 1106 bp fragment from pET28_AtSBPase was cloned in pNRSD_PcpcB_Km digested in the same way. | This work |
| pGLNN_PcpcB_PsFBPase | Ap Nat | Plasmid expressing PsFBPase gene under the control of P_cpcB_. A *Xba*I- *Xho*I 1211 bp fragment from pAMC1000 was cloned in pGLNN_PcpcB_Nat digested in the same way. | This work |
| pSLR2094_Ery | Ap, Ery | *slr2094* inactivation plasmid | This work |
| pARSB_PcpcB_Sp | Ap, Sp/St |  | This work |
| pET28-trxf1 | Km | TrxF1 expressing plasmid fused to Nt his tag | F.J. Cejudo Lab |
| pARSB_PcpcB_trxF1_Sp | Ap Sp/St | Plasmid expressing trxF1 gene under the control of P_cpcB_. A *Xba*I- *Xho*I 900 bp fragment from pET28-trxf1 was cloned in pARSB_PcpcB_Sp digested in the same way. | This work |
| pAM1000 | Ap | PsFBPase expressing plasmid fused to Nt his tag | M. Saharawy lab |
| pSLR0952::Sp | Ap Sp | *Slr0952* inactivation plasmid | This work |
