# Supplementary material for: Back to the future: Transplanting the chloroplast TrxF–FBPase–SBPase redox system to cyanobacteria
Source: Front Plant Sci. 2022 Nov 28;13:1052019. doi: 10.3389/fpls.2022.1052019 (PMC9742560; doi:10.3389/fpls.2022.1052019)
Supplement: Supplementary file 2 [file Presentation_1.pptx]

## Slide 1
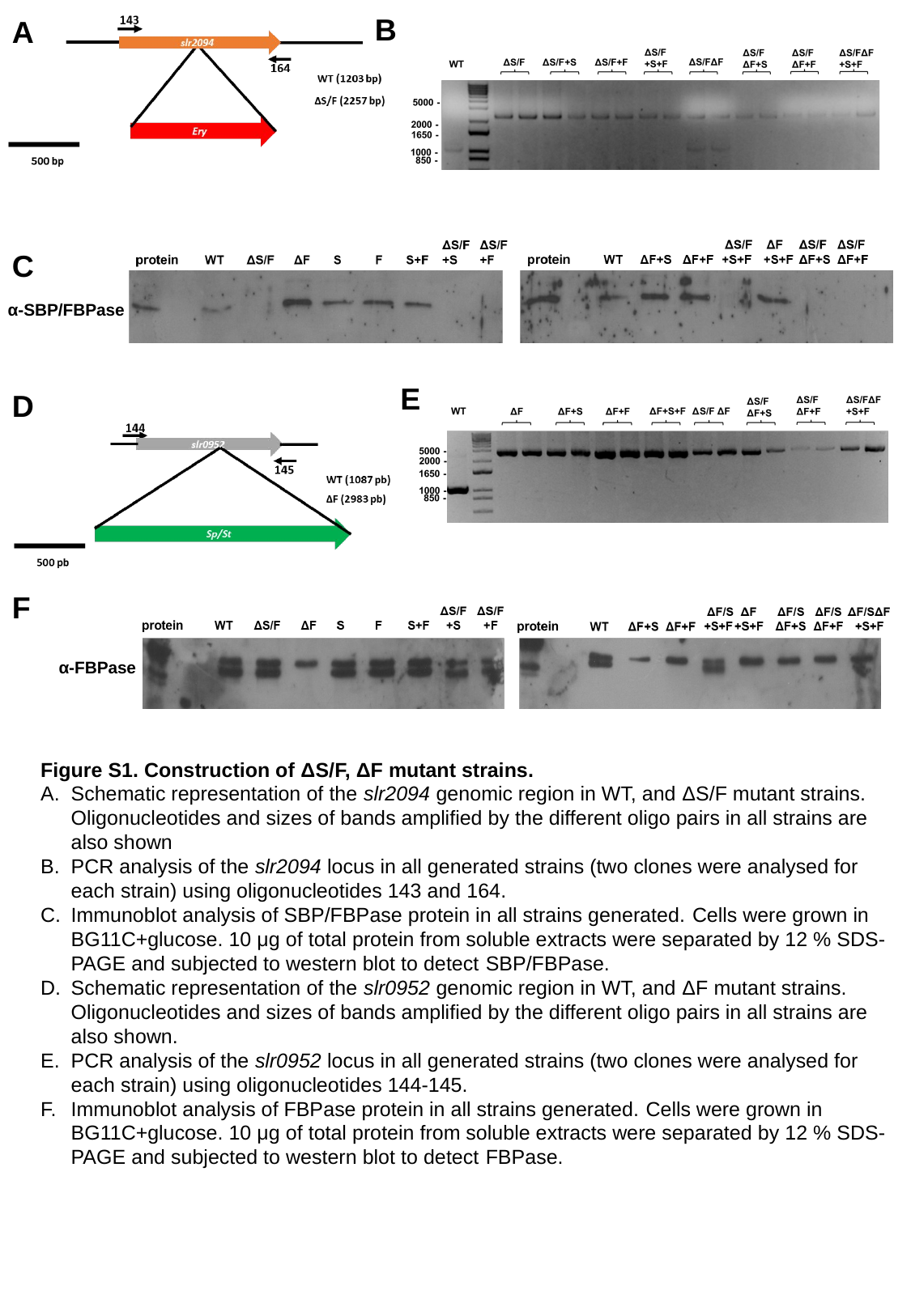

B
A
C
α-SBP/FBPase
E
D
F
α-FBPase
Figure S1. Construction of ΔS/F, ΔF mutant strains.
Schematic representation of the slr2094 genomic region in WT, and ΔS/F mutant strains. Oligonucleotides and sizes of bands amplified by the different oligo pairs in all strains are also shown
PCR analysis of the slr2094 locus in all generated strains (two clones were analysed for each strain) using oligonucleotides 143 and 164.
Immunoblot analysis of SBP/FBPase protein in all strains generated. Cells were grown in BG11C+glucose. 10 μg of total protein from soluble extracts were separated by 12 % SDS-PAGE and subjected to western blot to detect SBP/FBPase.
Schematic representation of the slr0952 genomic region in WT, and ΔF mutant strains. Oligonucleotides and sizes of bands amplified by the different oligo pairs in all strains are also shown.
PCR analysis of the slr0952 locus in all generated strains (two clones were analysed for each strain) using oligonucleotides 144-145.
Immunoblot analysis of FBPase protein in all strains generated. Cells were grown in BG11C+glucose. 10 μg of total protein from soluble extracts were separated by 12 % SDS-PAGE and subjected to western blot to detect FBPase.

## Slide 2
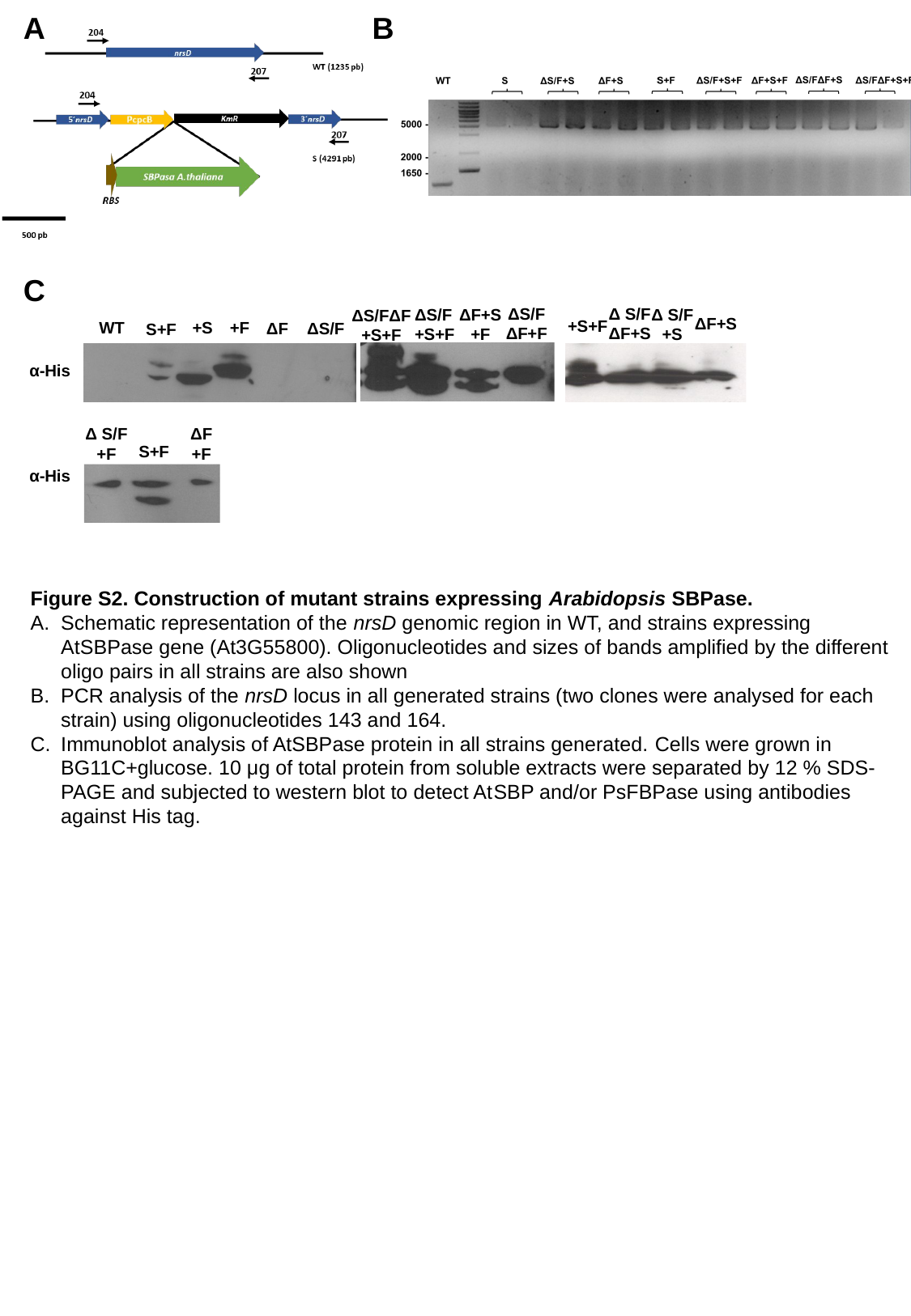

A
B
C
ΔS/F
ΔF+F
Δ S/F
ΔF+S
ΔS/F
+S+F
ΔF+S
+F
Δ S/F
+S
ΔS/FΔF
+S+F
ΔF+S
+S+F
+F
WT
+S
ΔS/F
ΔF
S+F
α-His
ΔF
+F
Δ S/F
+F
S+F
α-His
Figure S2. Construction of mutant strains expressing Arabidopsis SBPase.
Schematic representation of the nrsD genomic region in WT, and strains expressing AtSBPase gene (At3G55800). Oligonucleotides and sizes of bands amplified by the different oligo pairs in all strains are also shown
PCR analysis of the nrsD locus in all generated strains (two clones were analysed for each strain) using oligonucleotides 143 and 164.
Immunoblot analysis of AtSBPase protein in all strains generated. Cells were grown in BG11C+glucose. 10 μg of total protein from soluble extracts were separated by 12 % SDS-PAGE and subjected to western blot to detect AtSBP and/or PsFBPase using antibodies against His tag.

## Slide 3
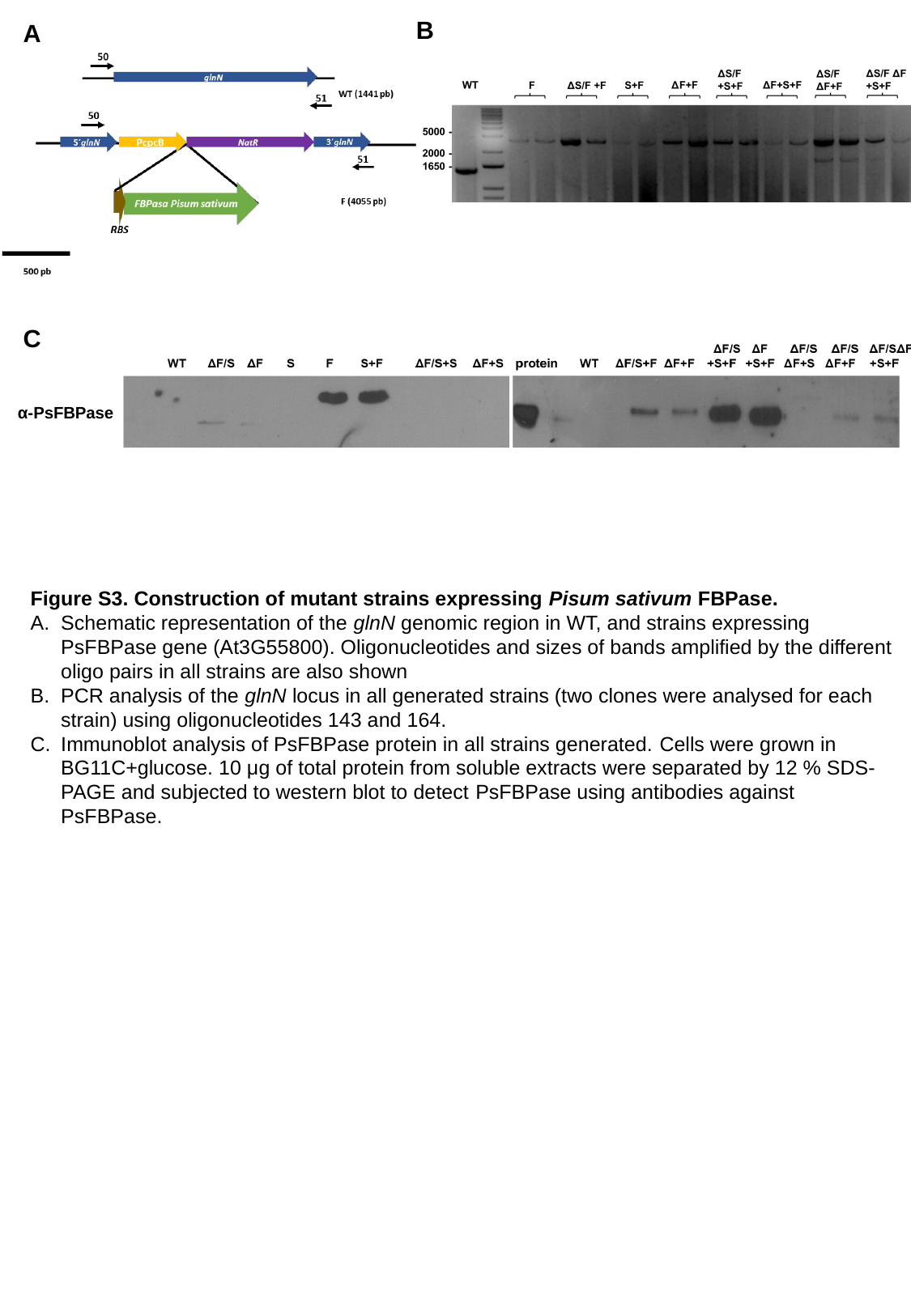

B
A
C
α-PsFBPase
Figure S3. Construction of mutant strains expressing Pisum sativum FBPase.
Schematic representation of the glnN genomic region in WT, and strains expressing PsFBPase gene (At3G55800). Oligonucleotides and sizes of bands amplified by the different oligo pairs in all strains are also shown
PCR analysis of the glnN locus in all generated strains (two clones were analysed for each strain) using oligonucleotides 143 and 164.
Immunoblot analysis of PsFBPase protein in all strains generated. Cells were grown in BG11C+glucose. 10 μg of total protein from soluble extracts were separated by 12 % SDS-PAGE and subjected to western blot to detect PsFBPase using antibodies against PsFBPase.

## Slide 4
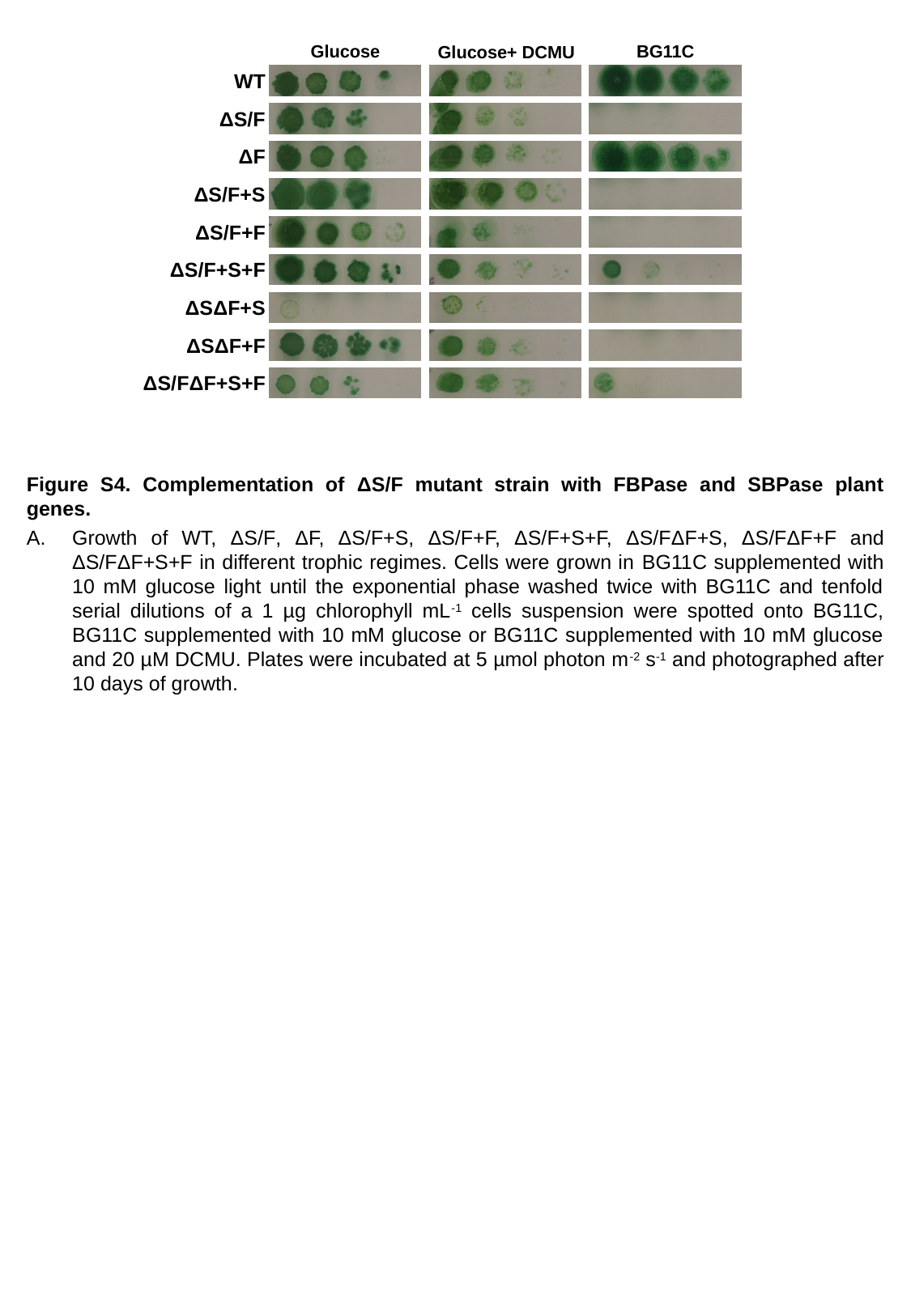

Glucose
BG11C
Glucose+ DCMU
WT
ΔS/F
ΔF
ΔS/F+S
ΔS/F+F
ΔS/F+S+F
ΔSΔF+S
ΔSΔF+F
ΔS/FΔF+S+F
Figure S4. Complementation of ΔS/F mutant strain with FBPase and SBPase plant genes.
Growth of WT, ΔS/F, ΔF, ΔS/F+S, ΔS/F+F, ΔS/F+S+F, ΔS/FΔF+S, ΔS/FΔF+F and ΔS/FΔF+S+F in different trophic regimes. Cells were grown in BG11C supplemented with 10 mM glucose light until the exponential phase washed twice with BG11C and tenfold serial dilutions of a 1 µg chlorophyll mL-1 cells suspension were spotted onto BG11C, BG11C supplemented with 10 mM glucose or BG11C supplemented with 10 mM glucose and 20 µM DCMU. Plates were incubated at 5 µmol photon m-2 s-1 and photographed after 10 days of growth.

## Slide 5
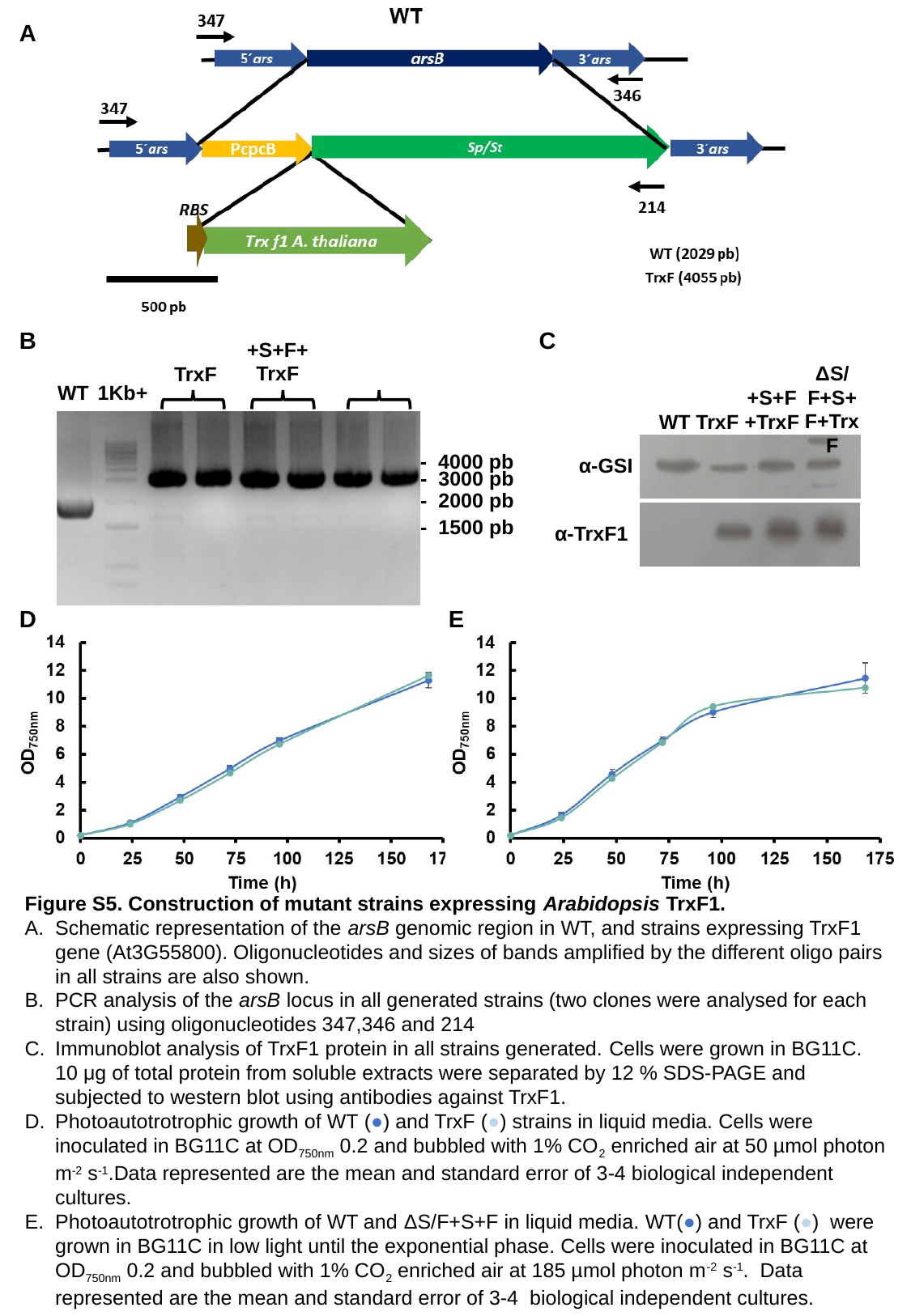

A
B
C
+S+F+TrxF
TrxF
WT
1Kb+
- 4000 pb
- 3000 pb
- 2000 pb
- 1500 pb
ΔS/F+S+F+TrxF
+S+F+TrxF
WT
TrxF
α-GSI
α-TrxF1
D
E
Figure S5. Construction of mutant strains expressing Arabidopsis TrxF1.
Schematic representation of the arsB genomic region in WT, and strains expressing TrxF1 gene (At3G55800). Oligonucleotides and sizes of bands amplified by the different oligo pairs in all strains are also shown.
PCR analysis of the arsB locus in all generated strains (two clones were analysed for each strain) using oligonucleotides 347,346 and 214
Immunoblot analysis of TrxF1 protein in all strains generated. Cells were grown in BG11C. 10 μg of total protein from soluble extracts were separated by 12 % SDS-PAGE and subjected to western blot using antibodies against TrxF1.
Photoautotrotrophic growth of WT (●) and TrxF (●) strains in liquid media. Cells were inoculated in BG11C at OD750nm 0.2 and bubbled with 1% CO2 enriched air at 50 µmol photon m-2 s-1.Data represented are the mean and standard error of 3-4 biological independent cultures.
Photoautotrotrophic growth of WT and ΔS/F+S+F in liquid media. WT(●) and TrxF (●) were grown in BG11C in low light until the exponential phase. Cells were inoculated in BG11C at OD750nm 0.2 and bubbled with 1% CO2 enriched air at 185 µmol photon m-2 s-1. Data represented are the mean and standard error of 3-4 biological independent cultures.

## Slide 6
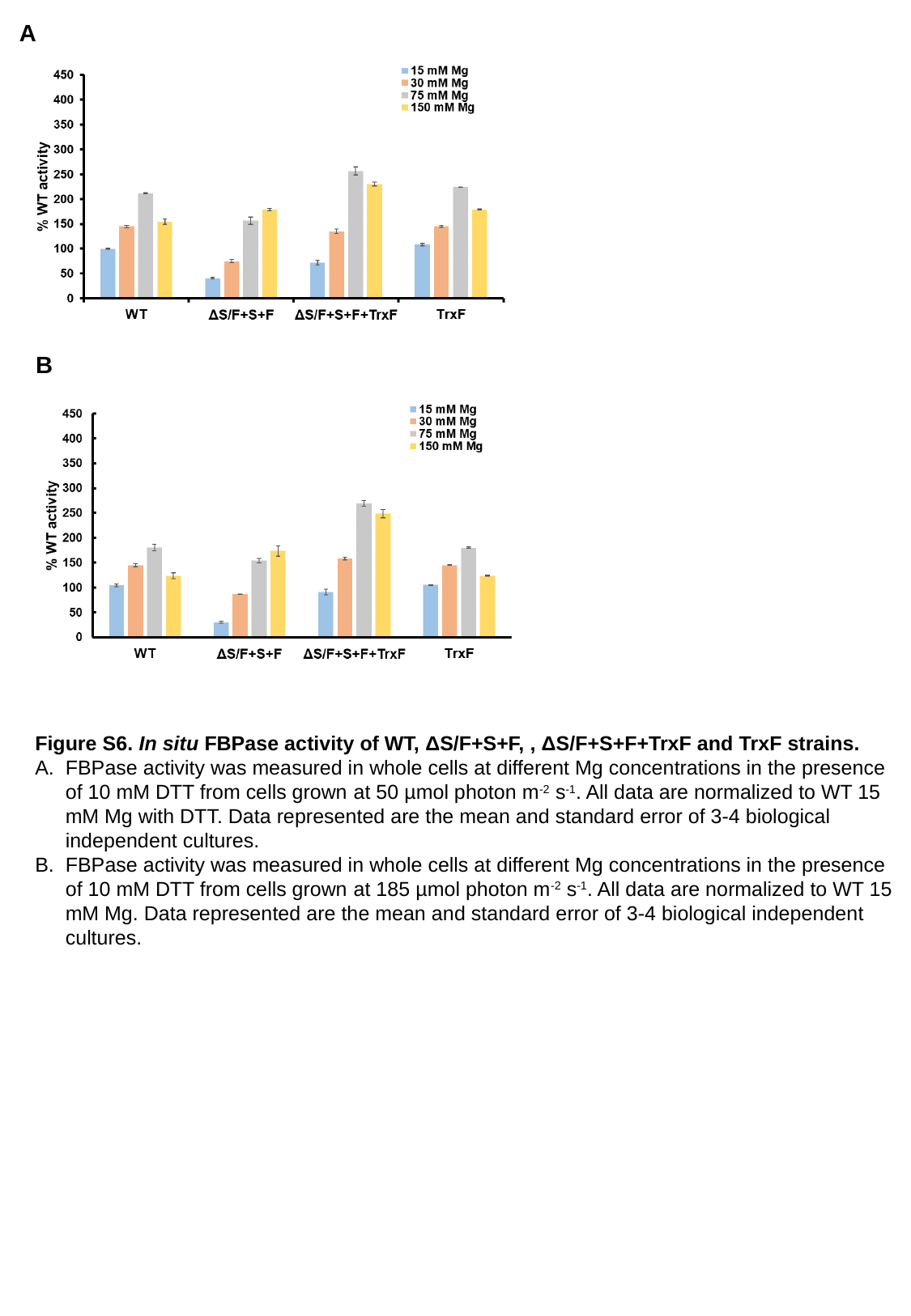

A
B
Figure S6. In situ FBPase activity of WT, ΔS/F+S+F, , ΔS/F+S+F+TrxF and TrxF strains.
FBPase activity was measured in whole cells at different Mg concentrations in the presence of 10 mM DTT from cells grown at 50 µmol photon m-2 s-1. All data are normalized to WT 15 mM Mg with DTT. Data represented are the mean and standard error of 3-4 biological independent cultures.
FBPase activity was measured in whole cells at different Mg concentrations in the presence of 10 mM DTT from cells grown at 185 µmol photon m-2 s-1. All data are normalized to WT 15 mM Mg. Data represented are the mean and standard error of 3-4 biological independent cultures.

## Slide 7
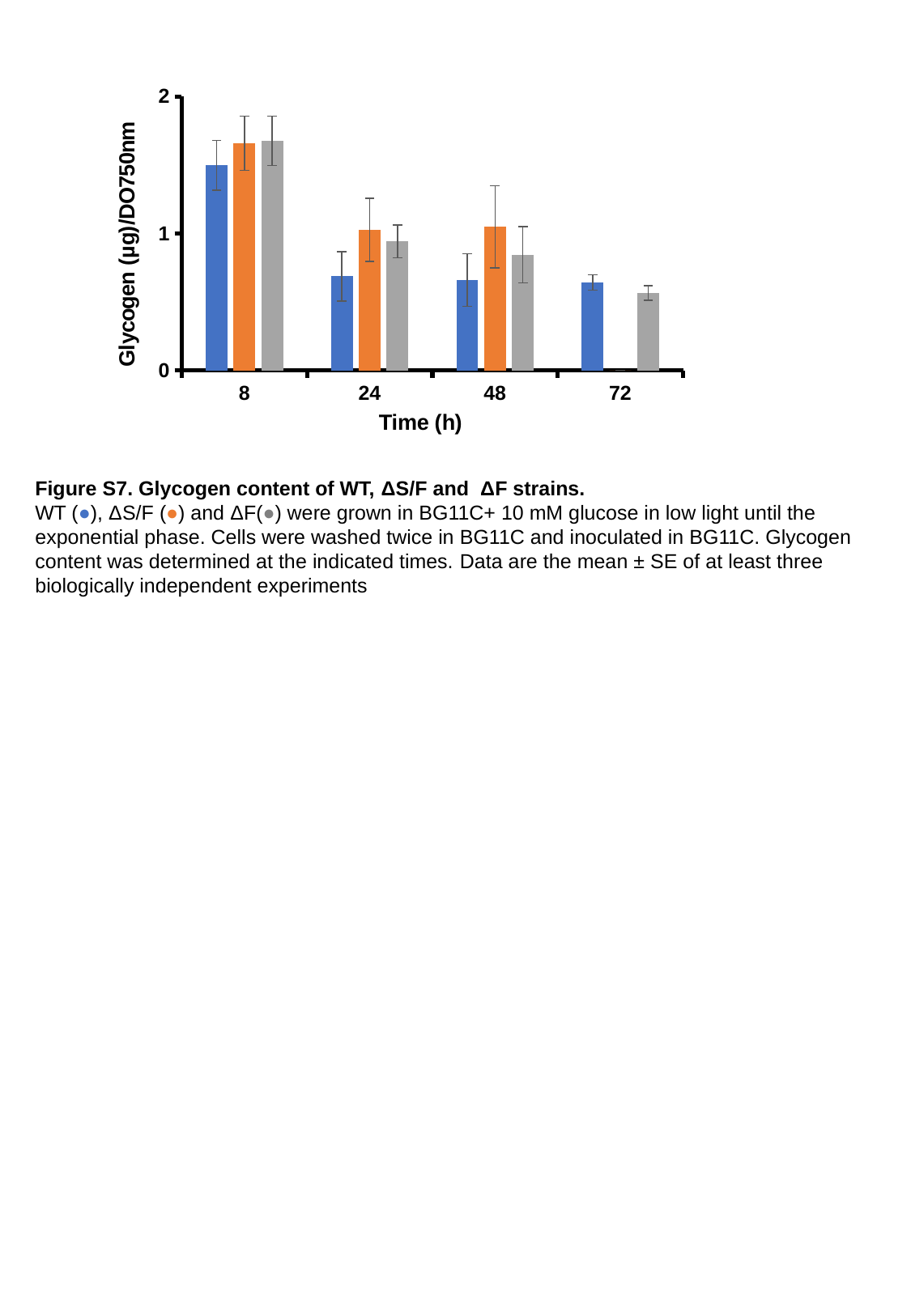

### Chart
| Category | | ΔF/S | ΔF |
|---|---|---|---|
| 8 | 1.4971755299586627 | 1.6580621187446902 | 1.6773411247388834 |
| 24 | 0.6867610216105255 | 1.0253623385097734 | 0.9423250492390955 |
| 48 | 0.6594564695280214 | 1.0474949397625666 | 0.8443091625455097 |
| 72 | 0.6418311466500475 | 0.0 | 0.5660103891593189 |Figure S7. Glycogen content of WT, ΔS/F and ΔF strains.
WT (●), ΔS/F (●) and ΔF(●) were grown in BG11C+ 10 mM glucose in low light until the exponential phase. Cells were washed twice in BG11C and inoculated in BG11C. Glycogen content was determined at the indicated times. Data are the mean ± SE of at least three biologically independent experiments
